# An intervention to manage compassion fatigue in oncology nurses in Durban, South Africa

**DOI:** 10.4102/hsag.v28i0.2376

**Published:** 2023-12-27

**Authors:** Dorien L. Wentzel, Anthony Collins, Petra Brysiewicz

**Affiliations:** 1 School of Nursing and Public Health, University of KwaZulu-Natal, Durban, South Africa; 2 Department of Social Inquiry, Faculty of Humanities and Social Sciences, La Trobe University, Melbourne, Australia; 3 Department of Psychology, Faculty of Arts, Rhodes University, Makhanda, South Africa

**Keywords:** compassion fatigue, compassion satisfaction, oncology nurses, self-care, intervention

## Abstract

**Background:**

Oncology nurses are involved through the often protracted and potentially traumatic continuum of diagnosis and treatment of their patients, which places them at high risk of developing compassion fatigue.

**Aim:**

The aim of the study was to develop and implement an in-facility intervention to manage compassion fatigue among oncology nurses in Durban, South Africa.

**Setting:**

The study was conducted with oncology nurses at state, private (private health insurance) and non-governmental oncology facilities (Hospice).

**Methods:**

The Self-Care Intervention for Oncology Nurses was developed and implemented using action research with a mixed methods sequential explanatory design. It involved an integrative review, Professional Quality of Life (ProQOL) v 5 questionnaires (*n* = 83) and in-depth individual interviews (*n* = 8).

**Results:**

Developed from the findings of the integrative review, quantitative and qualitative data, the Self-Care Intervention for Oncology Nurses comprised three components, namely psycho-education on risks (booklet), practices of remembrance (remembrance tree) and support structures (support group and follow-up family call). Overall, the participants enjoyed reading the booklet and engaging in the support group. There were varied responses to the remembrance tree and hesitancy to partaking in the follow-up phone call.

**Conclusion:**

The developed intervention could encourage awareness of compassion fatigue amongst oncology nurses’ engagement in self-care practices such as symbolic remembrance of patients and recognition of the value of support structures.

**Contribution:**

The intervention may assist oncology nurses in the provision of compassionate caring for their patients and potentially minimise compassion fatigue.

## Introduction

Compassion and empathy are viewed as important qualities relevant to nurses. Empathy can be a factor in job satisfaction; however, this same factor may result in harmful emotional and physical fatigue (Jarrad & Hammad 2020). Emotional fatigue can progress to compassion fatigue, as the nurses become emotionally impacted by the patients they are caring for (Boyle 2015; Fetter 2012; Figley 2005). Compassion fatigue is explained as the ‘cost of caring’ and is associated with having to witness pain and suffering (Figley 2001).

Because of compassion fatigue, nurses may emotionally detach, distancing themselves from their patients to manage their feelings (Ondrejková & Halamová 2022). However, distancing from patients may place the nurse at further risk of increasing the compassion fatigue and other psychological morbidities as the nurse is attempting to protect themselves emotionally, instead of coming to terms with challenging emotions (Ondrejková & Halamová 2022; Well-English, Giese & Price 2019). This detachment also risks reducing compassion satisfaction, the positive emotional reward of caring for patients that is a key protective factor against compassion fatigue. Literature details the long-term negative effects of compassion fatigue, which influences well-being, health, family life and work performance (Reiser & Gonzalez 2020; Stoewen 2020; Yilmaz, Ustun & Gunusen 2018). Adverse effects of compassion fatigue can cause institutional effects of increase in staff turnover and absenteeism, decrease in quality of patient care and satisfaction and decrease in patient safety, where the nurse may make mistakes by administering incorrect medications and fail to observe the deteriorating condition of patients (Blackburn et al. 2020; McClelland, Gabriel & DePuccio 2018).

Professional expectations of nurses include integrity and objectivity, which may be challenged by the emotional demands of work (ICN 2012). This causes tension, as nursing is a medically and emotionally caring profession, where nurses are expected to maintain care and empathy towards their patients while suppressing negative emotions of frustration and hopelessness (Well-English et al. 2019).

Stressors that oncology nurses face daily include complex painful treatments for patients, administration of unsuccessful treatments, traumatic emergencies, witnessing patient suffering, end-of-life care, death anxiety and the cumulative loss of the lives of patients with whom the nurse had a long-standing affiliation. Specific organisational difficulties such as high patient acuity, end-of-life care, decreased staff numbers and a lack of resources all compound the stress that the oncology nurse encounters (Sullivan et al. 2019). In a busy resource-constrained oncology ward, there is little time for supportive communication and debriefing for the oncology nurses, which might otherwise lessen the emotional effects experienced (Moghadam, Nasiri & Mahmoudirad 2022).

Oncology nurses need to be knowledgeable and clinically competent, to be able to empathetically assist cancer patients who themselves are feeling helpless and distressed (Blackburn et al. 2020). The high levels of stress experienced by oncology nurses can result in increased risk of inadequate self-care and decreased self-confidence (Traeger et al. 2013). Oncology nurses are at high risk of developing compassion fatigue, therefore familiarising nurses with the risks, warning signs and coping mechanisms and developing strategies to assist in the clinical area is essential to assist in coping with compassion fatigue (Potter et al. 2013). An extensive literature review was unable to locate any literature on psychosocial interventions for nurses working in oncology hospitals in South Africa. The international literature reviewed revealed that interventions of this type were all conducted in Western countries and, in single institutions, with small sample sizes (Wentzel & Brysiewicz 2017).

The aim of the study was to develop and implement an in-facility intervention to manage compassion fatigue in oncology nurses in Durban, South Africa.

## Methods

### Study design

The Self-Care Intervention for Oncology Nurses (SCION) was developed using action research with a mixed methods sequential explanatory design (Koshy, Koshy & Waterman 2011), (see Table 1). The study began with a quantitative component that displayed the extent of compassion fatigue experienced by oncology nurses. The findings from the quantitative data guided the qualitative component, which consisted of interviews to further explore factors that contribute to and protect against compassion fatigue (Creswell & Plano Clark 2011).

**TABLE 1:** Overview of the research process.

Research cycles	Objective of the cycle	Sample	Setting
One	Focus Group Discussions to establish the need for the proposed study.	Three FGDs held with oncology nurses (*n* = 16)	All three settings (State, private and NGO).
	Integrative review: To evaluate the effectiveness, Feasibility and nurses’ experiences of interventions to manage compassion fatigue.	Systematic search of Cochrane Library, JBI Library, DARE, CINAHL, MEDLINE, PubMed, EBSCO Host, SABINET, PsychINFO and Google Scholar.	Electronic search of literature published from 1992 to 2015 was performed.
Two	Analyse compassion fatigue, burnout and compassion satisfaction in nurses practising in oncology departments in Durban, South Africa.	Oncology nurses (registered and enrolled) completed ProQOL questionnaires (*n* = 83)	All three settings (State, private and NGO).
	Describe compassion fatigue from the perspective of oncology nurses in Durban, South Africa.	Individual interviews conducted with oncology nurses (*n* = 8)	All three settings (State, private and NGO)
Three	To develop an intervention by combining and comparing data obtained from the integrative review, quantitative and qualitative data.	Research team, oncology nurses, psychologist and PhD psychology student (*n* = 6)	Two workshops held
Four	Evaluation of the implementation of the evaluation – 4 weeks after implementation.	Two, 45-min focus groups were conducted to assess the implementation of the intervention (*n* = 8)	The intervention was conducted in two of the settings – Private and State setting

FGD, Focus Group Discussions; NGO, nonprofit organisation; ProQOL, Professional Quality of Life.

### Study setting

The research was conducted in three settings all providing oncology and palliative care in one municipality in KwaZulu-Natal, South Africa, namely a private hospital, a state hospital and a non-governmental hospice. These three settings depict the continuum of care for the oncology patient from diagnosis to palliative care. The SCION was implemented in two settings, namely a state hospital and a private hospital.

### Data collection process

This complex intervention was guided by the New Medical Research Council Guidance framework (Craig et al. 2008) and was developed from the synthesised evidence collected. The researcher initially held focus group discussions with oncology nurses in the three settings in order to explore the need for the study. All research participants were nurses (either professional nurses registered with the South African Nursing Council or enrolled nurses) currently working in any of the research settings for a minimum of 6 months. Nurses who had been working for less than 6 months in oncology were excluded from the study.

Guided by the action research approach (Koshy et al. 2011), a five-member research team comprising professional nurses employed at the three research settings was established and worked collaboratively with the researchers to help guide all aspects of the study. The conceptual framework guiding the study was the Compassion Fatigue Process (Figley 2001), which outlined the various factors that can predispose a person to develop compassion fatigue, namely exposure to a client, concern for the client, empathetic ability, self-regulation, compassion satisfaction, traumatic memories, prolonged exposure to clients and new life stressors. This model has been widely used globally and is the model from which the Professional Quality of Life (ProQOL) questionnaire was formulated (Stamm 2005). The existing evidence in the research area was gathered from an integrative review (Wentzel & Brysiewicz 2017) and quantitative data on oncology nurses’ compassion fatigue, compassion satisfaction and burnout (Wentzel & Brysiewicz 2018), as well as qualitative interviews with individual oncology nurses (Wentzel Brysiewicz & Collins 2019) were conducted. The complex intervention was developed by the researchers in conjunction with the research team, using this data and focused on strategies to manage compassion fatigue in oncology nurses.

Thereafter, workshops were held at two of the research sites, in conjunction with the research team, to present the intervention and to ask for feedback. Individual discussions with six experts (one psychologist, four oncology nurses and one PhD psychology student) were also conducted. Suggestions for the intervention were made and these included more detail to be included in the booklet as well as a psychologist to conduct the support groups. The intervention was then implemented in two settings over a period of 6 weeks.

Four weeks after the implementation, individual interviews were conducted with two unit managers and one oncology nurse, as well as two 30 min to 45 min focus group discussions in two different settings (private and state) to explore how useful, feasible, practical and appropriate the implementation of the intervention was.

### Data analysis

Analysis of the integrative review involved independent article appraisal from two reviewers. There were no disagreements; hence, a third reviewer was not needed. With the assistance of a statistician and using Stata v.13 statistical software, descriptive and inferential statistics were generated from the quantitative data. Means and standard deviation were calculated for the sub-scores for compassion fatigue, burnout and compassion satisfaction. Qualitative data were analysed using manifest content analysis (whereby meaning units were then condensed; following this, codes were developed, which were then grouped into categories (Erlingsson & X 2017; Graneheim & Lundman 2003). The development of the complex intervention was guided by The New Medical Research Council Guidance, MRC (Craig et al. 2008) and the conceptual framework for the study. The key elements of this framework include development, feasibility, implementation and evaluation of the intervention. The participants validated the findings via email and in-person communication. In the mixing of the quantitative and qualitative data, as well as the input from experts and the research team, triangulation was achieved providing depth and clarity (Fielding 2012).

### Rigour in a mixed method study

ProQOL v5 comprised three different scales, where each scale is psychometrically unique and cannot be combined with other scores. Cronbach’s alpha reliabilities for the three scales are as follows: compassion fatigue alpha = 0.80, burnout alpha = 0.72 and compassion satisfaction alpha = 0.89. Professional Quality of Life has been reported in over 200 peer-reviewed articles, thereby demonstrating well-established construct validity (Stamm 2005).

The credibility of the qualitative data were upheld by prolonged engagement with the settings, research team and participants (2 years). An audit trail was compiled by the researcher, thereby maintaining dependability. Prior to the study commencing the researcher bracketed her bias. All verbatim transcriptions, member checks and validity checks were recorded thus demonstrating the authenticity of the data (Guba & Lincoln 1994).

Rigour was achieved in this mixed method study, as the researchers provided the reader insight into how the study was conducted by providing detailed methodology (Creswell, Fetters & Ivankova 2004).

### Ethical considerations

Ethical approval was granted by BREC ethics committee of the University of KwaZulu-Natal (BF 140/14), and permission was obtained from the three settings. Given the sensitive nature of the study, the researcher (DW) was aware of the importance of protecting vulnerable participants and thus negotiated with employment assistance programmes in the settings to ensure support for participants should the need arise during the study. Written informed consent was obtained, and participants were given assurance of confidentiality, voluntary participation and their freedom to withdraw from the study at any time. Pseudonyms in the interviews and focus groups were assigned by the researcher. At the beginning of the study, the ownership of the data and the research team members’ role in this study were discussed and agreed upon.

## Results

### Intervention development

**FIGURE 1:**
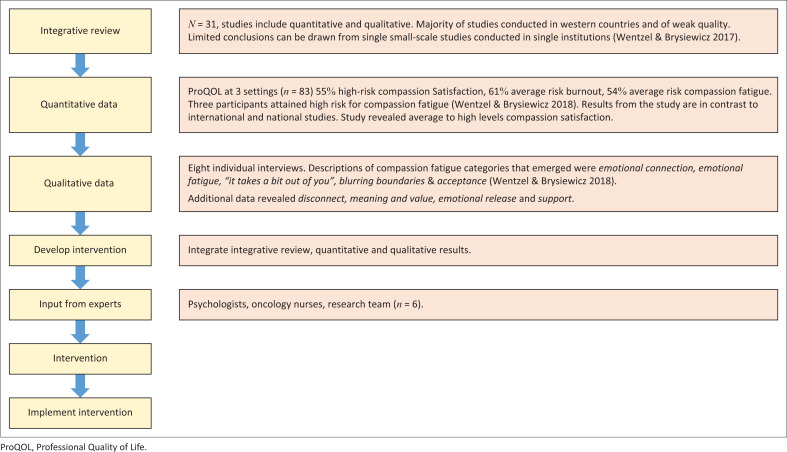
Study design process and development of intervention.

From the findings of the four cycles, the SCION draft version was developed (see Table 1 and Figure 1).

This complex intervention comprised three components, namely: (1) psycho-education, (2) remembrance and (3) support structures (see Table 2).

**TABLE 2:** Self-care interventions for oncology nurses.

Component	Component details	Details of component
Psycho-education	Booklet 6 pages Educational information, self-care and support regarding compassion fatigue.	Definition, signs and symptoms of compassion fatigue. Self-reflection, self-care, risk accumulation and coping activities.
Remembrance	Remembrance tree: A wire baobab tree was set up in an accessible place where it was visible to staff and patients. Together with small cards and pegs to attach the messages.	When a patient dies, staff can write a note about the patient and peg onto the tree. This process can help staff to grieve, remember the close patient relationship and assist with starting the closure process.
	Family phone call: A staff member phones relatives 4–6- weeks after a loved one has died to assess how they are coping.	The phone call can provide support helping family members in their grieving and closing process. This phone call can also assist the staff member in his or her own grieving and closure for the patient.
Support	Clinical psychologist hospital based: Came weekly × 2 sessions, thereby allowing participants to discuss and reflect on experiences and how these experiences may have affected them.	Guidance on coping skills to promote normalisation thereby allowing nurses to recognise that it is normal to have these symptoms and reactions. An attempt to address both psychological and social needs of nurses while providing a safe environment for nurses. Clinical psychologists will also encourage the nurses to carry on with the process through the formation of peer-support groups.
	Peer support, to promote the formation of a support group whereby nurses could discuss their fears and concerns and reflect on their experiences and the effect these may have had on them.	Promote peer support so that self-supporting and self-disclosing becomes an automatic, self-sustaining process.

#### Psycho-education

Quantitative and qualitative data demonstrated a need to ensure these nurses were provided with educational information, self-care and support regarding compassion fatigue. A booklet was developed highlighting the definition, signs and symptoms of compassion fatigue, the importance of self-reflection and awareness of being at risk (including accumulating risk over time), as well as the value of deliberate self-care and activities to promote prevent and cope with compassion fatigue.

#### Remembrance

Remembrance of patients is an important step in the grieving process; as the emotional transition is one in which many nurses feel isolated (Madsen et al. 2022). Participants voiced it as important to address the act of remembering their patients and to assist in managing repetitive emotional loss for nurses who were emotionally connected with their patients:

- Remembrance tree: Using a remembrance tree (a small wire tree) to symbolise the relationship and the loss of a patient gives nurses an opportunity to reminisce and express their respect, and hopefully, attain finality and peace (Fetter 2012; Vaclavik, Staffileno & Carlson 2018). Fetter (2012) suggests a remembrance tree to promote remembrance that can be utilised as an emotional strategy that can assist in managing repetitive loss.
- Family phone call: This involved phoning relatives 4–6 weeks after their loved one had died to ask how they were coping. This phone call could assist family members in their grieving and closure process and assist the staff member in his or her own grieving and closure for the patient.

#### Support structures

The qualitative data highlighted actions that could be useful in attempting to address both the psychological and social needs of the nurses, while providing a safe environment for nurses to express their experiences and fears. This guided the decision to include formal support from a clinical psychologist, as follows:

- Clinical Psychologist: The support group was established to allow nurses to discuss their fears and experiences, as well as how these experiences may have affected them. Two sessions per setting were held. These offered guidance from the clinical psychologist in the form of coping skills to promote normalisation of emotional responses, thereby allowing people to recognise that it is usual to have these symptoms and reactions. Clinical psychologists also encouraged the nurses to carry on with this process through the development of a formal peer support group.
- Peer support: Promote sustainable peer support among the nurses in such a way that disclosure and mutual support becomes an automatic self-sustaining process.

### Evaluating the implementation of the intervention

The participants were asked if there were any advantages or disadvantages in the implementation of the intervention.

Participants commented that the booklet was ‘Easy to read and understand’, and that it ‘Explained the differences between burnout and compassion fatigue’ and ‘Made us think of caring for ourselves’. The clinical psychologist commented that the content in the booklet ‘provided the nurses with the chance to review, to reflect and to share their understanding of compassion fatigue’. Participants stated that the support group was helpful and ‘It was good to have someone we could talk to and express ourselves’ and ‘listen to our fears and feelings’.

There was hesitancy from both settings regarding the follow-up phone call to the loved ones of deceased patients. Participants stated they would not feel ‘comfortable speaking as they wouldn’t know what to say’ and questioned that by phoning the relatives they felt ‘They were admitting guilt over the patients’ death’.

After the remembrance tree was established in the two settings, the researcher made unannounced visits to see the acceptance of the remembrance tree. In one setting, the tree was placed on the duty room desk, so that it could be seen by staff and patients while in the other setting the tree was placed out of sight, away in the Comfort Room, for fear of ‘disturbing patients by reinforcing their mortality’. There were, however, varied responses between the two settings regarding the use of the remembrance tree. At the state hospital, participants eagerly contributed to writing messages for patients who had died; however, there was reluctance from participants at the private institution to do so. When asked why there were no messages on the tree a senior nurse replied, ‘We have not had many deaths, therefore we have not used the tree’.

## Discussion

Educational programmes are often the first step towards introducing structured experiences that can enhance worker’s understanding of the need for self-awareness about compassion fatigue and improve knowledge about healthy lifestyle adjustments that can aid recovery at work and outside work (Poulsen et al., 2015; Sullivan et al. 2019; Vu & Bodenmann 2017). Potter et al. (2013) concur that participants in their study reported that they acquired positive coping skills from their education, which then enabled them to deal with behavioural evasion. Interventions assisted in oncology nurses embracing and sustaining healthy self-care practices, which could increase resilience (Blackburn et al. 2010). Well-English et al. (2019) concur that a proactive intervention could assist nurse managers in the prevention and early identification of compassion fatigue in their employees, with early referral to employment assistance programmes or psychosocial support. Effective strategies for prevention, self-reflection and early identification of compassion fatigue make it possible for oncology nurses to continue with extended compassionate and professional caring for oncology patients under challenging conditions (Fukumori et al. 2020; Sullivan et al. 2019; Zajac, Moran & Groh 2017).

In the interviews of this study, participants fondly remembered their patients and described the emotional connection they had made with these patients (Wentzel & Brysiewicz 2018). They also recounted the emotional loss they felt when a patient’s condition deteriorated and how they managed this by detaching themselves from patients and becoming numb (Wentzel & Brysiewicz 2018). Remembering patients by writing messages, which are then displayed together with group remembrance sessions, has been shown to provide a supportive setting whereby nurses could freely vent their feelings (Sullivan et al. 2019).

In this study, the wire baobab remembrance tree elicited different responses from the two settings. One setting embraced the tree and it was full of messages remembering patients who had died, while the other setting had no messages on the remembrance tree. Remembrance of patients is an important step in the grieving process as this emotional transition is one in which many nurses feel isolated (Madsen et al. 2022). The remembrance tree symbolises loss and offers nurses an opportunity to reminisce and express their respect (Fetter 2012).

Well-English et al. (2019) reinforce that peer support networks held at regular sessions could assist nurses to speak openly and express their feelings together while listening to other nurses’ experiences. Informal and formal debriefing sessions can ease the emotional burden and promote peer support (Blackburn et al. 2020; Vu & Bondenmann 2017). ‘Invisible caring’, whereby nurses reflect on their own patient care and competence, could assist in the facilitation of closure of patients who have died whilst simultaneously building resilience (Olling et al. 2021).

Consideration to one’s self-care and professional well-being can greatly influence patient care; however, this area is often ignored by nurses (Vu & Bodenmann 2017). Reasons for non-acceptance of self-care practices could also include workplace cultures and individual beliefs (Wentzel & Brysiewicz 2018). Huynh (2022) reiterates additional support to prevent and recognise early compassion fatigue, especially since the coronavirus disease 2019 (COVID-19) pandemic.

## Limitations

The study was conducted in one area in KwaZulu-Natal; therefore, the findings cannot be generalised. The sample was homogenous as all participants were female. All the nurses working in the two settings received education booklets; however, only those on duty when the psychologist conducted the sessions received the psychological support. The researcher was known to some of the participants and this may have influenced participants to offer socially acceptable responses. The outcome of the SCION intervention was not evaluated.

## Implications for practice

Institutional management should be aware of the risk of their oncology nurses developing compassion fatigue. It is therefore recommended that strategies are in place to address this and that group sessions be facilitated by a mental health professional to help oncology nurses to explore and make sense of personal work experience, promote emotional well-being and increase resilience (Blackburn et al. 2020).

Findings from the study demonstrate the need to equip nurses with knowledge and skills to recognise and manage compassion fatigue. As shown by the varying responses to the remembrance tree, interventions should be appropriate to specific settings. Nurses should be able to design self-care plans and strive for compassion satisfaction. Institutions should provide regular professional education programmes, thereby assisting nurses to cope with the emotional demands of nursing (Sullivan et al. 2019; Ramalisa, Du Plessis & Koen 2018).

Further research is proposed to evaluate if this intervention is effective, feasible and sustainable in oncology and other specialised areas where nurses practice.

## Conclusion

The SCION intervention can prompt oncology nurses to be more aware of compassion fatigue, to engage in self-care practices such as symbolic remembrance of patients and to recognise the value of support structures. The intervention could assist nurse managers in the prevention and early identification of compassion fatigue in their employees with early referral to Employment Assistance Programmes and Psychosocial support).

Despite the challenging conditions oncology nurses endure, there are strategies for early identification and management of compassion fatigue so that nurses can continue to provide compassionate professional care to their patients.
